# The effect of bilateral intrapleural infusion of lidocaine with fentanyl versus only lidocaine in relieving pain after coronary artery bypasses surgery

**DOI:** 10.12669/pjms.331.10847

**Published:** 2017

**Authors:** Kamran Shadvar, Sarvin Sanaie, Ata Mahmoodpoor, Mitra Safarpoor, Bahman Nagipour

**Affiliations:** 1 Kamran Shadvar, Assistant Professor of Anesthesiology, Fellowship of Critical Care Medicine, Cardiovascular Research Center, Tabriz University of Medical Sciences, Tabriz, Iran; 2 Sarvin Sanaie, Assistant Professor of Nutrition, Lung Disease and Tuberculosis Research Center, Tabriz University of Medical Sciences, Tabriz, Iran; 3 Ata Mahmoodpoor, Associate Professor of Anesthesiology, Fellowship of Critical Care Medicine, Department of Anesthesiology, Tabriz University of Medical Sciences, Tabriz, Iran; 4 Mitra Safarpoor, General Physician, Student Research Center, Tabriz University of Medical Sciences, Tabriz, Iran; 5 Bahman Nagipour, Assistant Professor of Anesthesiology, Fellowship of cardiac Anesthesia, Department of Anesthesiology, Tabriz University of Medical Sciences, Tabriz, Iran

**Keywords:** Intra-pleural analgesia, Fentanyl, Lidocaine, CABG

## Abstract

**Background and Objective:**

Pain control during surgery in order to cause analgesia and reduce the somatic and autonomic response may decrease the morbidity. Intrapleural catheter embedding during surgery under direct vision of surgeon is safe and easy and without potential risk of thoracic epidural block. The aim of this study was to investigate the effect of bilateral intrapleural infusion of lidocaine with fentanyl versus only lidocaine in relieving pain after coronary artery bypass surgery.

**Methods:**

In this prospective randomized double blind clinical trial,130 adult patients undergoing elective CABG with age range of 20 to 60 years were divided into two groups receiving either lidocaine and fentanyl (group A) or lidocaine (group B). The analgesia was evaluated every two hours in all intubated and non-intubated patients using Visual analog scale (VAS) and data were analyzed using SPSS software package.

**Results:**

Of all patients, 67 (51.5%) were males and 63 (48.5%) were females. The average age of subjects was 53.49 ± 5.099 years. Mean pain score six hours after the surgery was statistically different between the groups at all times.

**Conclusion:**

The pain in patients receiving combination of lidocaine and fentanyl is less than patients receiving only lidocaine.

## INTRODUCTION

Pain causes severe autonomic and physiological responses which can be very dangerous in patients with ischemic heart disease.^1^ Pain control during surgery in order to cause analgesia and reduce the somatic and autonomic response decreases the morbidity.^2^ A variety of strategies including spinal anesthesia, intercostal nerve block, systemic opioids, and non-steroidal anti-inflammatory drugs and alpha-2 agonist are used to control pain.^3^ There is debate about the effectiveness of various types of preventive analgesia; but the combination of local anesthesia and general anesthesia may improve pain control after surgery.^4^ An effective analgesia protocol improves lung function and allows physiotherapy to start sooner after the surgery.^5^ The use of systemic opioids can cause respiratory depression and bowel dysfunction.^3^ Spinal anesthesia is the most effective way to control pain after thoracotomy, but carries a significant risk of epidural hematoma and respiratory depression.^6^ The disadvantages of peripheral nerve block includes high concentrations of plasma level of drug due to high blood feeding around intercostal nerves, patients discomfort and prolonged neuralgia.^7^

Local anesthesia through intrapleural catheter is another way to control pain. Intrapleural anesthesia was first described by Reiestad.^8^ Previous studies showed that spinal and intrapleural anesthesia were effective and safe in patients undergoing cardiac surgery with minimal invasion.^9^

The intrapleural block in the supine position leads to lower thoracic ganglion block but cervical ganglion and lumbar network are not blocked thus hemodynamics during painful stimuli like thoracotomy remains relatively stable.^10^

Bupivacaine is studied for its effect on the relief of pain after thoracic surgery. There are few reports of side effects associated with its use.^9^ However, with the discovery of opioid receptors localized in peripheral tissues, attentions were drawn to the environmental impact of opioid through intrapleural infusion.^11^ Intrapleural bupivacaine is widely used after thoracic surgery. Although most of the studies show good results, there are conflicting results on its effectiveness.^12^

Due to different techniques of coronary artery bypass graft surgery (CABG) which differ the location and severity of pain in this group and as to the best of our knowledge, there is no study about pain control levels using intrapleural block after CABG, we decided to conduct a study to compare the effect of intrapleural lidocaine and fentanyl with only lidocaine in reducing pain after CABG.

## METHODS

This prospective randomized double blind clinical trial study was conducted in Tabriz Shahid Madani Hospital, after obtaining approval of the university ethics committee and written consent from patients. This study was registered in IRCT with the registration number of: IRCT2015112714728N2. One hundred thirty adult patients between the age of 20 to 60 years and undergoing elective CABG having 3 to 6 hours operation time were divided into two groups.

### Inclusion and Exclusion Criteria

Inclusion criteria were EF≥30%, Cr ≤ 1.8, elective CABG surgery, age of 20 to 60 years. Exclusion criteria were previous pleural disease, COPD, Diabetic neuropathy, seizures, allergy to local anesthesia, drug addiction, drug habits to analgesics, calcium channel blockers, alpha2-agonists, history of previous heart surgery and surgery complications such as Aortic dissection, redo operation due to postoperative bleeding, high drainage after surgery (more than 1000 ml/24h).

All patients received oral lorazepam (1mg) the night before surgery, and morphine (0.1mg/kg IM) and promethazine (0.5mg/kg IM) half an hour before going to the operating room. Induction of anesthesia was done using intravenous midazolam (0.1mg/kg), fentanyl (5μg/kg), atracurium (0.15mg/kg), lidocaine (1mg/kg) and maintenance of anesthesia by using TIVA method (Total intravenous anesthesia) with a combination of midazolam, fentanyl and atracurium. All patients were monitored under standard technique during surgery. In the later stage of operation sterile white suction catheter was bilaterally inserted to intrapleural space from the embedded chest tube site. To avoid interference of injection and discharging possible bleeding of the patient, and in order to prevent disrupting injection process when removing the Chest tube medication was not injected through Chest tube. After injection of intraplural drugs, Chest tube was clamped temporarily for 10 minutes to prevent the medication discharge. Long time clamping was avoided in order to prevent possible disorders in the process of unloading mediastinal bleeding in patients.

Patients were divided into two groups receiving either lidocaine and fentanyl (group A) (65 patients) or lidocaine (group B) (65 people). At the end of the surgery and in the ICU, 20 ml of lidocaine 0.75% + fentanyl 2mcg/kg for group A and 20 ml of lidocaine 0.75% in Group B were injected by an anesthesiologist through the intraplural catheter every 6 hours (The dose of lidocaine was 150 mg/6h in each group and total dose 600 mg in 24 hours and the dose of fentanyl was 120 mcg/6h and total dose 720 mcg in group A. The total volume of injected drugs in intraplural space was the same. According to the maximum dose of lidocaine which is 5mg/kg per injection and according to the half-life of lidocaine which is 1.5 to 2 hours there were no problem in terms of toxicity (inotropic and anticonvulsants drugs were ready available near the patient for the treatment of emergency conditions caused by complications). The analgesia was evaluated in all intubated and non-intubated patients using Visual analog scale (VAS) every two hours after the patient awaking and data were analyzed using SPSS software. If the VAS was 5 or more, intravenous morphine was injected at dose of 0.5mg/kg/IV. The VAS and the type and total amount of used analgesics were recorded.

The data were analyzed using the statistical software program Statistical Package for the Social Sciences Version 16.0, SPSS. Kolmogorov–Smirnov test was used to assess the normality of the variables distribution. The data were analyzed using Chi-square test to compare parametric qualitative variables and Independent *t*-tests to compare parametric quantitative variables between two groups. Differences in variables during the study were assessed with a repeated measures analysis of variance. *P*<0.05 was considered as significant for all statistical tests.

## RESULTS

There was no significant difference in terms of demographic characteristics including age, sex and BMI between the two groups. (Table-I)

**Table-I:** Demographic Data of the patients in both groups.

	Group A	Group B	P value
Age (years)	54.48±4.657	52.51±5.362	0.247
Sex			0.862
Male (%)	52.3	50.8	
Female (%)	47.7	49.2	
BMI	27.72±1.941	27.75±1.976	0.938

BMI: Body mass index

There were no significant differences between two groups in terms of time of surgery, CPB time, the aortic cross clamping time, EF, number of grafts, duration of mechanical ventilation, duration of stay in the ICU, MAP (before anesthesia and 2, 6, 12, 18 and 24 hours after surgery), HR (before anesthesia and 2, 6, 12, 18 and 24 hours after surgery), PH, PaO_2_ and PCO_2_ (at 6, 12, 18 and 24 hours after surgery). There was not a significant difference in mean SpO_2_ average before anesthesia and at 2, 6, 12, 18 hours after surgery but SpO_2_ 24 hours after surgery was statistically different between two groups. (Table-II)

**Table-II:** Perioperative variables of the patients in both groups.

	Group A	Group B	P value
Operation time (min)	288.15±13.953	287.16±16.1	0.707
Pump time (min)	110.46±8.155	112.26±8.024	0.207
Aortic Clamp time (min)	77.2±11.041	78.46±11.405	0.291
EF (mean %0)	45.48±6.479	46.05±4.389	0.559
Graft numbers (mean)	3.26±0.442	3.37±1.038	0.448
Duration of mechanical ventilation (hours)	11.03±4.885	10.64±3.282	0.597
Longs of ICU stay (days)	3.08±0.375	3.05±0.28	0.578
Spo2 before operation (%)	91.57±2.704	91.52±2.278	0.916
MAP before operation (mmHg)	75.34±6.188	74.14±5.193	0.233
Heart rate before operation (beat/min)	94.74±8.822	95.33±8.346	0.697
Spo2 in ICU (%)	98.94±1.014	98.97±1.045	0.865
MAP in ICU (mmHg)	71.23±4.404	70.63±4.278	0.432
HR in ICU (beat/min)	68.88±5.795	87.98±5.175	0.255

EF: Ejection fraction, MAP: Mean arterial pressure, HR: Heart rate

Average pain score of patients in two-hour intervals after surgery is listed in Table-III. Pain scores at all measured times after surgery except 6 hours after surgery had statistically significant difference between two groups and was less in group A (group receiving lidocaine with fentanyl) than group B (lidocaine alone group).

**Table-III:** Mean pain score with VAS at different times after surgery in two groups.

Hours	Group A	Group B	P value
2	3.389±0.359	4.18±0.497	0.000
4	4.25±0.531	4.83±0.417	0.000
6	4.22±0.573	4.45±0.662	0.054
8	3.91±0.491	4.62±0.7	0.000
10	3.84±0.407	4.19±0.393	0.000
12	3.63±0.517	4.09±0.423	0.000
14	3.59±0.526	3.98±0.381	0.000
16	3.58±0.558	3.89±0.475	0.003
18	3.57±0.499	3.85±0.592	0.031
20	3.27±0.479	3.72±0.487	0.000
22	3.22±0.417	3.56±0.531	0.001
24	3.15±0.364	3.43±0.585	0.006

VAS: Visual analog scale.

The average dose of analgesic medication in subjects was 6.771±3.305. This amount in group A and B was 3.91±1.147 and 8.61±2.907 respectively. Average dose of oral analgesic in the subjects was 1240.25±595.319. This amount in group A and B were 764.189±497.721 and 1519/841±455.392, respectively. There was no side effect in any patient.

## DISCUSSION

Post thoracotomy pain is one of the most severe pains that a patient experiences in perioperative period, which can continue for months and even years. Inadequate control of pain impairs breathing and effective coughing, which leads to atelectasis and pulmonary complications, including infection,^5^ insufficient removing of secretions and mucus adhesion and impaired gas exchange.^7^,^13^ Pain after cardiac surgery is a debatable clinical issue that can increase morbidity and mortality. Respiratory activity rapidly worsens and slowly heals after cardiac surgery.^14^ In this study, patients undergoing elective CABG were divided into two groups. The group A received intra-pleural fentanyl with lidocaine and the group B received intra-pleural lidocaine. Variables such as pain were compared and measured according to VAS at different times for both groups.

Ramajoli et al. observed that in patients suffering from pleuritic chest pain caused by benign or malignant disease, intrapleural block relatively stables the blood hemodynamic status within a painful stimulus during thoracotomy.^10^

Karakaya et al in their study found that intrapleural bupivacaine and fentanyl combined with epinephrine have more analgesic effect compared to only bupivacaine.^15^ In our study, in the group receiving lidocaine with fentanyl, mean pain score based on VAS at all measured time points (except 6 hours after surgery) is lower than the group receiving only lidocaine. This could mean that adding fentanyl to analgesic treatment regimen can reduce the pain after thoracotomy; but further studies are needed to confirm this.

In the study of Pennefather et al of 68 patients undergoing thoracotomy, 39 patients were randomly assigned to two groups receiving either bupivacaine 0.25% (40 mg) intrapleural with epinephrine 1: 200,000 or saline. There was no statistical difference in VAS score between the two groups. They concluded that intrapleural bupivacaine does not produce adequate analgesia for shoulder pain control after unilateral thoracotomy^16^ but in our study, mean pain score based on VAS in all measured time points (except 6 hours after surgery) was less than the control group.

In the study of Raffin et al, 14 patients were under analgesics treatment with intrapleural lidocaine 2% and 16 patients were in the control group. They concluded that intra-pleural analgesia with lidocaine after thoracotomy has poor results. The mean VAS score in the group that received lidocaine after receiving bolus was 6.6.^17^ Although there was no control group receiving placebo in our study, even the group receiving only lidocaine also has good analgesic effect. In our study, the mean VAS score in the group receiving just lidocaine in two hours after surgery was 4.18. Elman et al observed insufficient analgesic effect of intrapleural bupivacaine after posterolateral thoracotomy.^18^

Kambam et al. demonstrated that intrapleural anesthesia improves pain control in patients undergoing thoracotomy with posterior and lateral incision.^19^ In addition, Oka et al showed that continuous infusion of thoracic epidural bupivacaine reduces incidence of Supraventricular tachycardia after lung resection surgery.^20^ Ogus et al reported improved lung function was related with reduced pain levels after surgery in patients who received intrapleural bupivacaine.^21^

D’Andrilli et al examined pain relief after intrapleural-intercostal block after surgery in patients who were under mini-thoracotomy for pulmonary resection and concluded that in the group receiving bupivacaine pain, mortality, morbidity and duration of hospitalization is significantly less than the control group.^22^ In this study, in 12 and 24 hours after operation, pain was lower in the group receiving bupivacaine. In our study the mean pain score based on VAS in all measured time points (other than 6 hours after surgery) was less than the control group. Aykac et al found that, patients who received intravenous morphine had higher level of morphine in plasma and more side effects as compared to those who received intrapleural morphine.^7^ Previous studies have showed that the intrapleural side effects of morphine are very low. Teik observed greater analgesic effect of intrapleural bupivacaine compared to saline.^23^ In contrast, several studies have reported limited effectiveness of this method^19^,^24^ which may be due to drug dilution in the pleural space and drug drainage through chest tubes may lead to less drug concentration at the site of the receptors and insufficient analgesic effect.

Mashaqi et al. examined 40 patients who underwent CABG with sternotomy in two groups either intrapleural injection of 2% lidocaine or placebo and found that intrapleural lidocaine is a safe method to reduce chest tube related pain and improving lung function after CABG.^25^ This study is in line with our survey but we didn’t use pulmonary function test to find the improvement of lung function in two groups.

### Limitations of the Study

It is the lack of data in use of LIMA (left internal mammary artery) for CABG between the two groups. Given that harvesting LIMA from inside the chest wall may be more intense pain than no harvest after operation.

## CONCLUSIONS

Intrapleural lidocaine with fentanyl can more effectively suppresses pain compared to lidocaine alone in patients ndergoing elective CABG surgery. We recommend conducting further studies with more sample size, examining pain in the long term in two groups.
